# Pediatric lymphoproliferative disorders – Emerging insights and management: A narrative review

**DOI:** 10.1097/MD.0000000000047367

**Published:** 2026-01-23

**Authors:** Emmanuel Ifeanyi Obeagu

**Affiliations:** a Department of Biomedical Laboratory Science, Africa University, Mutare, Zimbabwe.

**Keywords:** Epstein–Barr virus, immune dysregulation, immunodeficiency, pediatric lymphoproliferative disorders, targeted therapy

## Abstract

Pediatric lymphoproliferative disorders (PLPDs) encompass a spectrum of conditions marked by the abnormal proliferation of lymphocytes, often linked to genetic mutations, immune dysregulation, and infectious agents like Epstein–Barr virus. These disorders present with varied clinical phenotypes, ranging from benign lymphadenopathy to severe, life-threatening malignancies. Recent advancements in molecular diagnostics and imaging have significantly enhanced the early identification and classification of PLPDs, paving the way for timely and effective interventions. Understanding the pathophysiological mechanisms underlying PLPDs has also been instrumental in guiding the development of novel therapeutic strategies. The management of PLPDs has evolved with the advent of targeted therapies, including monoclonal antibodies and small molecule inhibitors, which have demonstrated promising efficacy in mitigating disease progression. For severe or refractory cases, hematopoietic stem cell transplantation remains a curative option, especially for disorders associated with primary immunodeficiencies. Despite these advancements, challenges persist in ensuring equitable access to advanced diagnostics and therapies, particularly in resource-limited settings. Furthermore, optimizing treatment regimens to minimize long-term complications and improve quality of life remains a critical area of focus.

## 1. Introduction

Pediatric lymphoproliferative disorders (PLPDs) are a heterogeneous group of conditions characterized by the uncontrolled proliferation of lymphocytes, frequently arising due to genetic, immunological, and environmental factors. These disorders can manifest in various forms, ranging from benign reactive processes to severe malignancies such as lymphoma. The diversity in their clinical presentation and pathophysiological underpinnings poses significant challenges in their diagnosis and management.^[1]^ One of the primary drivers of PLPDs is immune dysregulation, often rooted in primary immunodeficiency syndromes. Disorders such as X-linked lymphoproliferative syndrome (caused by mutations in the SH2D1A gene) and autoimmune lymphoproliferative syndrome (linked to Fas/FasL pathway defects) highlight the genetic basis of many PLPDs. Additionally, chronic viral infections, particularly those caused by Epstein–Barr virus (EBV), play a pivotal role in the pathogenesis of certain LPDs. EBV’s ability to immortalize B lymphocytes and evade immune surveillance often contributes to the unchecked proliferation characteristic of these conditions.^[2]^ The clinical spectrum of PLPDs is remarkably broad, encompassing asymptomatic cases identified incidentally to life-threatening complications involving systemic organ dysfunction. Persistent lymphadenopathy, recurrent infections, and systemic symptoms such as fever and weight loss are among the hallmark features. In severe cases, PLPDs may progress to malignancies, emphasizing the importance of early diagnosis and intervention. Such variability necessitates a tailored approach to patient evaluation and management, incorporating a detailed clinical history, laboratory findings, and advanced imaging studies.^[3]^

Advancements in diagnostic technologies have significantly improved the understanding and classification of PLPDs. Tools such as flow cytometry, next-generation sequencing (NGS), and positron emission tomography-computed tomography (PET-CT) imaging enable clinicians to identify underlying genetic mutations, assess lymphocyte phenotypes, and evaluate disease extent with unprecedented precision. Furthermore, these technologies facilitate the differentiation of PLPDs from other hematological or infectious conditions, ensuring appropriate therapeutic interventions.^[4]^ In parallel with diagnostic advancements, therapeutic strategies for PLPDs have evolved considerably. Targeted therapies, such as monoclonal antibodies like rituximab, and small molecule inhibitors, including JAK inhibitors, have emerged as effective options for managing specific subtypes of PLPDs. Immunomodulatory agents such as sirolimus have demonstrated promise in addressing the immune dysregulation underlying many of these disorders. For severe or refractory cases, hematopoietic stem cell transplantation remains a curative option, particularly in patients with genetic or primary immune defects.^[1]^

## 2. Aim

This review aims to provide an overview of the pathophysiology, diagnostic advances, and therapeutic strategies for PLPDs, with an emphasis on emerging insights and challenges in their management.

## 3. Rationale

Pediatric LPDs stand at the intersection of immunology, oncology, and genetics, offering a compelling window into the interplay of these disciplines in young, vulnerable patients. Unlike adults, where immune systems are fully developed, children’s immune responses are still maturing, making them uniquely susceptible to disorders that involve abnormal lymphoid cell proliferation. These disorders can range from transient, infection-related conditions to life-threatening malignancies, requiring a nuanced understanding of their underlying causes and management. The increasing prevalence and recognition of LPDs in pediatric populations, particularly those linked to genetic mutations or viral infections such as EBV, underscore the urgency for targeted research. Innovations in diagnostic tools, like NGS and advanced imaging techniques, have begun to unravel the complexities of these disorders. Similarly, therapeutic advancements, including immunotherapy and gene-editing technologies, hold promise for transforming outcomes in these patients.^[1–4]^ The rationale for a comprehensive review of pediatric LPDs lies in bridging knowledge gaps across these areas. By synthesizing insights into the etiology, classification, and cutting-edge treatment modalities, we can provide a roadmap for clinicians and researchers to better address the unique challenges posed by these disorders. Ultimately, understanding pediatric LPDs not only illuminates the pathophysiology of lymphoproliferative diseases but also contributes to broader advancements in pediatric medicine and immune-related therapies. This review seeks to spotlight the progress made so far, the hurdles that remain, and the future directions that could shape the management of pediatric LPDs, with an overarching aim of improving survival and quality of life for affected children.

## 4. Review methodology

This review article was developed following a structured and systematic methodology to ensure comprehensive and accurate synthesis of the available literature on pediatric LPDs. The process involved the following steps.

## 5. Research objectives

The primary objective of this review was to analyze and consolidate emerging insights into the etiology, classification, diagnostic advancements, and management strategies of pediatric LPDs. This included a focus on genetic, immunological, and viral factors influencing disease pathogenesis, as well as novel therapeutic approaches.

## 6. Literature search strategy

A detailed literature search was conducted using electronic databases, including PubMed, Scopus, Web of Science, and Google Scholar. The search was carried out from [starting date] to [end date]. Keywords and Medical Subject Headings terms were employed to ensure comprehensive retrieval of relevant studies. Key terms included:

- *“Pediatric LPDs”*
- *“Lymphoma in children”*
- *“Genetic predisposition in lymphoproliferative diseases”*
- *“EBV in pediatric oncology”*
- *“Immunotherapy for LPDs”*

Boolean operators (AND/OR) were used to refine the search and retrieve studies that specifically addressed the objectives of this review.

## 7. Inclusion and exclusion criteria

The following criteria were applied to select relevant articles:

- **Inclusion criteria:**
  ○ Peer-reviewed articles, clinical trials, meta-analyses, and systematic reviews.
  ○ Studies focusing on pediatric populations (≤18 years) with LPDs.
  ○ Articles published in English.
  ○ Studies addressing etiology, pathogenesis, diagnostic techniques, and treatment modalities.
- **Exclusion criteria:**
  ○ Articles focusing exclusively on adult populations.
  ○ Studies with insufficient data or unclear methodologies.
  ○ Conference abstracts and non-peer-reviewed literature.

## 8. Ethical approval

Not applicable.

## 9. Pathophysiology

The pathophysiology of PLPDs is rooted in a complex interplay of genetic, immunological, and environmental factors that drive the uncontrolled proliferation of lymphocytes. This dysregulation often stems from mutations affecting immune signaling pathways, particularly those involving T-cell and B-cell regulation, apoptosis, and immune surveillance. These abnormalities disrupt the delicate balance between lymphocyte activation, proliferation, and programmed cell death, leading to excessive or persistent lymphocyte expansion.^[3]^ Genetic mutations are a central contributor to PLPDs, especially in cases associated with primary immunodeficiencies. Mutations in genes such as SH2D1A (encoding the SAP protein) in X-linked lymphoproliferative syndrome or FAS in autoimmune lymphoproliferative syndrome disrupt normal immune function and predispose individuals to lymphocyte hyperproliferation. These genetic defects impair the immune system’s ability to regulate lymphocyte activation, resulting in chronic stimulation, autoimmunity, or malignant transformation.^[5]^ Viral infections, particularly EBV, play a pivotal role in the pathogenesis of many PLPDs. EBV infects B lymphocytes and establishes latency, enabling the virus to evade immune detection while driving cellular proliferation. In immunocompromised hosts, such as those with congenital immunodeficiencies or undergoing immunosuppressive therapy, the unchecked proliferation of EBV-infected lymphocytes may lead to LPDs or lymphoma. Chronic viral antigenic stimulation further exacerbates immune dysregulation, perpetuating lymphocyte expansion and increasing the risk of malignancy.^[4]^

Immune dysregulation is another hallmark of PLPDs, often characterized by an impaired balance between effector and regulatory lymphocytes. This imbalance leads to persistent antigen-driven proliferation and failure to terminate immune responses. Inflammatory cytokines, such as interleukin-6 and interferon-gamma, play a role in driving lymphocyte survival and proliferation. In some cases, defective apoptosis due to Fas–FasL pathway mutations or other intrinsic cellular abnormalities leads to prolonged lymphocyte lifespan, contributing to lymphadenopathy, organ infiltration, and autoimmune complications.^[6]^ Epigenetic modifications and the tumor microenvironment also influence the pathophysiology of PLPDs. Epigenetic alterations, such as DNA methylation and histone modification, can silence tumor suppressor genes or activate oncogenes, promoting lymphocyte transformation. Additionally, the tumor microenvironment, composed of immune cells, stromal cells, and cytokines, supports lymphocyte survival and proliferation while creating an immunosuppressive milieu that favors disease progression.^[7]^ In severe cases, PLPDs may progress to malignancies such as lymphoma. This progression is often driven by cumulative genetic mutations, chromosomal abnormalities, and sustained immune dysregulation. For example, EBV-positive LPDs may undergo malignant transformation through viral oncogene activation and host genome instability.^[8]^

## 10. Clinical presentation

PLPDs exhibit a wide spectrum of clinical manifestations, reflecting the diverse etiologies and underlying mechanisms of these conditions. Symptoms may range from mild, nonspecific findings to severe, life-threatening complications. The clinical presentation is often influenced by the underlying immunological or genetic abnormality, the presence of viral infections such as EBV, and the extent of lymphocyte proliferation or tissue infiltration.^[9,10]^ One of the most common presentations of PLPDs is persistent lymphadenopathy, characterized by swollen lymph nodes that do not resolve after infections have subsided. These nodes may be painless or tender and are often located in cervical, axillary, or inguinal regions. While benign causes like reactive lymphadenopathy are frequent in children, persistent or generalized lymphadenopathy raises suspicion for LPDs or malignant transformation.^[11,12]^ Systemic symptoms, including fever, night sweats, fatigue, and weight loss, are frequently observed in PLPDs. These symptoms, known collectively as “B symptoms,” are often indicative of an underlying malignant or inflammatory process. Children with PLPDs may also present with nonspecific complaints such as malaise, reduced appetite, or recurrent infections, reflecting immune dysregulation or bone marrow dysfunction.^[13]^

Organ involvement is a hallmark of more advanced or severe cases of PLPDs. Hepatosplenomegaly, resulting from lymphocyte infiltration in the liver and spleen, is a common finding. In some cases, organomegaly may cause abdominal distension, discomfort, or even impaired organ function. Bone marrow infiltration may lead to cytopenias, manifesting as anemia, thrombocytopenia, or leukopenia, which can predispose children to fatigue, bleeding, or infections.^[14,15]^ Autoimmune complications are frequently associated with PLPDs, particularly in the context of immune dysregulation syndromes. Examples include autoimmune hemolytic anemia, immune thrombocytopenic purpura, and autoimmune neutropenia, which can further complicate the clinical picture. These features may occur in isolation or as part of a broader autoimmune phenomenon, necessitating careful differentiation from other autoimmune disorders.^[16]^ Infections play a dual role in PLPDs, both as triggers and consequences of the disease. Persistent or recurrent infections, especially with opportunistic pathogens, may signal an underlying immunodeficiency or lymphoproliferative process. Infections with EBV are particularly notable, as they can precipitate or exacerbate lymphoproliferation and, in some cases, lead to life-threatening complications such as hemophagocytic lymphohistiocytosis.^[17]^ The risk of malignant transformation is a critical concern in PLPDs. Children with conditions such as X-linked lymphoproliferative syndrome or chronic active EBV infection are at heightened risk of developing lymphoma, particularly non-Hodgkin lymphoma (NHL) or Hodgkin lymphoma (HL). The progression to malignancy may be heralded by rapidly enlarging lymph nodes, worsening systemic symptoms, or the emergence of extranodal disease in sites such as the gastrointestinal tract, central nervous system, or bone marrow.^[18]^

## 11. Diagnostic advances

Advances in diagnostic methodologies have significantly improved the identification, classification, and management of PLPDs. These innovations enable early detection, precise diagnosis, and monitoring of disease progression, which are critical for tailoring effective treatment strategies.

### 11.1. Flow cytometry

Flow cytometry has become a cornerstone in the diagnostic evaluation of PLPDs. This technique facilitates the analysis of lymphocyte subsets and the identification of aberrant immunophenotypes, such as abnormal expression of surface markers on B or T cells. For instance, overexpression of CD10 or CD20 may indicate malignant B-cell proliferation, while loss of CD27 in T cells can suggest immune dysregulation. The use of multi-parameter flow cytometry allows for simultaneous evaluation of multiple markers, providing detailed insights into the cellular landscape of PLPDs.^[19–21]^

### 11.2. Molecular diagnostics

NGS and polymerase chain reaction (PCR)-based techniques have revolutionized the molecular diagnosis of PLPDs. NGS enables the identification of genetic mutations and translocations associated with these disorders, such as mutations in the SH2D1A or STAT3 genes. PCR-based assays are particularly valuable for detecting clonality in lymphocyte populations, aiding in distinguishing between reactive and neoplastic processes. The ability to analyze genetic and epigenetic changes enhances our understanding of the disease’s pathogenesis and informs targeted therapeutic approaches.^[2,22–24]^

### 11.3. Imaging modalities

Advanced imaging techniques have improved the assessment of disease burden and the detection of extranodal involvement in PLPDs. PET-CT is highly effective for visualizing metabolically active lesions and monitoring treatment responses. Magnetic resonance imaging offers superior soft-tissue contrast, making it particularly useful for detecting central nervous system involvement. The integration of imaging data with clinical and molecular findings facilitates a comprehensive evaluation of disease extent and activity.^[11,25,26]^

### 11.4. Viral load assays

Given the strong association of EBV with many forms of PLPDs, viral load quantification has become an essential diagnostic tool. Real-time PCR assays for EBV DNA in blood or tissue samples provide valuable information about viral activity and its role in driving lymphoproliferation. These assays are particularly critical in diagnosing EBV-associated conditions, such as chronic active EBV infection and EBV-positive lymphomas.^[27–29]^

### 11.5. Biopsy and histopathology

Lymph node or tissue biopsy remains a definitive diagnostic method for PLPDs. Histopathological examination provides insights into the architectural changes and cellular composition of affected tissues. Immunohistochemistry further aids in classifying LPDs by highlighting specific markers, such as CD30 for HL or CD20 for B-cell lymphomas. The combination of histological and molecular findings enhances diagnostic accuracy.^[2,29,30]^

### 11.6. Functional immunological assays

Functional assays, such as cytotoxic T-cell activity tests and cytokine profiling, provide additional layers of diagnostic detail. These tests evaluate the functional integrity of the immune system, particularly in cases where immune dysregulation plays a pivotal role. Abnormalities in cytokine production, such as elevated interleukin-6 or interferon-gamma levels, may correlate with disease activity or progression.^[31,32]^

### 11.7. Advances in liquid biopsy

Liquid biopsy, involving the analysis of circulating tumor DNA (ctDNA) or other biomarkers in blood, is an emerging diagnostic modality in PLPDs. This minimally invasive approach offers the potential for real-time monitoring of disease progression and response to therapy. Liquid biopsy is particularly promising for detecting minimal residual disease and early relapse.^[33,34]^

### 11.8. Artificial intelligence (AI) and machine learning

The integration of AI and machine learning algorithms in diagnostic workflows has begun to enhance the interpretation of complex datasets, including imaging, flow cytometry, and molecular profiles. AI-driven tools can identify subtle patterns and correlations, aiding in early diagnosis and personalized treatment planning.^[35,36]^

The combination of these diagnostic advancements has significantly improved the precision and efficiency of diagnosing PLPDs. Ongoing innovations in technology and methodology hold the promise of further enhancing our ability to understand and manage these challenging pediatric conditions.

## 12. Management strategies

Pediatric LPDs are a diverse group of conditions that involve the abnormal growth and proliferation of lymphocytes. These disorders can be benign, as in some reactive conditions, or malignant, as in various forms of lymphoma. The management of pediatric LPDs requires a comprehensive, multidisciplinary approach that incorporates chemotherapy, immunotherapy, radiation therapy, and supportive care. As the landscape of pediatric oncology continues to evolve, several emerging treatment strategies are reshaping the management of these complex conditions.

### 12.1. Chemotherapy

Chemotherapy has been the mainstay of treatment for many malignant LPDs in children, including HL and NHL. Standard regimens such as the cyclophosphamide, doxorubicin, vincristine, and prednisone regimen for NHL and adriamycin, bleomycin, vinblastine, and dacarbazine for HL are widely used and form the foundation of 1st-line treatment. For pediatric HL, chemotherapy is often used in combination with radiation therapy, particularly in early-stage disease. However, due to the significant long-term side effects of radiation, including increased risks for secondary cancers, the trend is moving toward using chemotherapy alone or at reduced doses. This reduction in radiation exposure is a particularly important strategy to minimize the risk of future malignancies in pediatric patients who are more susceptible to radiation-induced DNA damage. In cases of NHL, chemotherapy regimens like cyclophosphamide, doxorubicin, vincristine, and prednisone are tailored based on the subtype, stage, and risk factors of the disease. Advanced-stage NHL or those involving extranodal sites, such as the bone marrow or central nervous system, often require more intensive chemotherapy and longer treatment durations. The addition of rituximab, an anti-CD20 monoclonal antibody, has significantly improved outcomes in B-cell lymphomas, and this combination of chemotherapy and targeted therapy is now a standard approach in pediatric NHL management.^[37–39]^

### 12.2. Immunotherapy

The advent of immunotherapy has revolutionized the treatment of pediatric LPDs, particularly in cases that are refractory to traditional chemotherapy. Immune checkpoint inhibitors and chimeric antigen receptor T-cell (CAR-T) therapy are at the forefront of emerging treatments for pediatric lymphomas. Immune checkpoint inhibitors, such as pembrolizumab (anti-programmed cell death protein 1 [PD-1]) and nivolumab (anti-PD-1), have shown promising results in the treatment of relapsed or refractory HL. These therapies work by blocking the PD-1/programmed death-ligand 1 (PD-L1) pathway, which is often hijacked by tumors, including Reed–Sternberg cells in HL, to evade immune detection. By inhibiting this immune checkpoint, these drugs help to re-activate the immune system, allowing T-cells to recognize and attack cancerous cells. Clinical trials have demonstrated that pembrolizumab and nivolumab can lead to durable responses in children with relapsed HL, offering an alternative to traditional salvage therapies. CAR-T therapy is a cutting-edge treatment that involves genetically modifying a patient’s own T-cells to target and destroy cancer cells. For pediatric patients with relapsed or refractory B-cell lymphomas, CAR-T therapy targeting the CD19 antigen has become a game-changer. This therapy has led to remarkable remissions in patients who have exhausted other treatment options. However, CAR-T therapy is associated with significant risks, including cytokine release syndrome and neurotoxicity, which require careful monitoring and management. Despite these challenges, CAR-T therapy represents a new frontier in pediatric lymphoma treatment, particularly for those with refractory disease.^[40–42]^

### 12.3. Radiation therapy

Radiation therapy plays a critical role in the management of pediatric LPDs, particularly for localized lymphomas. In HL, radiation has traditionally been used in combination with chemotherapy, especially in early-stage disease. However, with advances in chemotherapy and the goal of reducing long-term side effects, radiation therapy is increasingly reserved for patients with bulky disease or those who do not respond to chemotherapy alone. In NHL, radiation is often used as part of the treatment for localized or bulky disease and may be combined with chemotherapy. However, the importance of minimizing radiation exposure, especially in pediatric patients, has prompted a shift toward lower doses or targeted radiation to reduce the risk of secondary cancers and other long-term effects. Newer techniques, such as proton therapy, offer the potential for more precise radiation delivery, sparing surrounding healthy tissues.^[43,44]^

### 12.4. Stem cell transplantation

Hematopoietic stem cell transplantation, also known as bone marrow transplantation, is considered in pediatric LPDs when the disease is refractory to chemotherapy and other treatments. Autologous stem cell transplantation, where the patient’s own stem cells are harvested, purified, and re-infused after high-dose chemotherapy, is commonly used in the treatment of lymphoma, particularly for relapsed or refractory B-cell lymphomas. Allogeneic stem cell transplantation, which involves using a donor’s stem cells, may be considered in cases where the patient’s lymphoma relapses after an autologous transplant or when there is an underlying immunodeficiency contributing to the lymphoproliferation. The procedure is highly complex and carries risks, including graft-versus-host disease, but it can offer curative potential in select patients.^[45–47]^

### 12.5. Supportive care

Supportive care is a critical aspect of the management of pediatric LPDs. Chemotherapy and other aggressive treatments can result in significant side effects, including neutropenia, anemia, thrombocytopenia, and infections. The risk of infections is particularly high during chemotherapy, so prophylactic antibiotics, antifungals, and antiviral medications are routinely used to prevent life-threatening infections. Pain management, nutritional support, and psychosocial care are essential to improve the quality of life for children undergoing treatment. Bone marrow suppression often requires blood product transfusions, including red blood cells and platelets, to manage anemia and thrombocytopenia. Furthermore, physical and emotional support, including counseling for the child and family, is crucial, as the challenges of coping with cancer and its treatment can have long-lasting psychological effects.^[41,48]^

### 12.6. Personalized medicine

One of the most exciting advancements in the management of pediatric LPDs is the concept of personalized medicine. Advances in molecular diagnostics, including NGS, allow for the identification of specific genetic mutations and alterations that drive the development of lymphoma. For example, mutations in the MYC, BCL-2, and TP53 genes have been linked to aggressive forms of lymphoma, and therapies targeting these mutations are in development. Personalized treatment plans that are based on the genetic profile of the patient’s disease enable clinicians to select the most effective therapy while minimizing unnecessary side effects. Additionally, ongoing research is focused on identifying biomarkers that can predict treatment response, resistance, and relapse, further optimizing treatment decisions and improving outcomes for pediatric patients.^[1,49,50]^

## 13. Emerging insights

Pediatric LPDs represent a diverse and evolving group of conditions, ranging from benign, reactive lymphoid proliferations to aggressive, malignant lymphomas. The understanding and treatment of these disorders have advanced significantly in recent years, driven by improved diagnostic tools, better understanding of disease biology, and the development of novel therapeutic approaches. This section highlights some of the key emerging insights that are shaping the future of pediatric LPDs.

### 13.1. Advances in molecular understanding of LPDs

One of the most significant advancements in the field of pediatric LPDs is the enhanced understanding of the molecular and genetic underpinnings of these diseases. The traditional approach to classifying LPDs focused largely on histopathological features. However, recent advances in genomics, including the use of NGS, have enabled researchers and clinicians to identify specific genetic mutations, chromosomal translocations, and molecular markers that drive the pathogenesis of pediatric LPDs. For example, in pediatric NHL, recurrent mutations in genes such as MYC, BCL-2, and TP53 have been identified, and these genetic alterations are associated with more aggressive forms of lymphoma. The recognition of such mutations has not only improved diagnostic accuracy but also paved the way for the development of targeted therapies aimed at these molecular drivers. In HL, the identification of genetic alterations in the immune microenvironment, particularly in the Reed–Sternberg cells, has revealed insights into how the tumor evades immune surveillance. Mutations in the JAK/STAT signaling pathway, for example, play a critical role in the pathogenesis of HL, making it an important target for new therapies. The use of molecular profiling to assess gene expression signatures and identify risk factors for relapse is becoming a key aspect of personalized treatment plans.^[51,52]^

### 13.2. Immunotherapy

Immunotherapy has emerged as one of the most promising and transformative approaches in the treatment of pediatric LPDs, especially for cases that are resistant to conventional therapy. The ability to harness the body’s own immune system to target and destroy cancerous cells has revolutionized the management of these disorders. One of the most exciting areas of immunotherapy involves immune checkpoint inhibitors, such as pembrolizumab and nivolumab. These agents work by blocking immune checkpoints like PD-1/PD-L1, which cancer cells exploit to evade immune detection. In pediatric HL, where the Reed–Sternberg cells often express PD-L1, immune checkpoint inhibitors have shown remarkable efficacy, especially in patients with relapsed or refractory disease. Clinical trials have demonstrated that these drugs can lead to durable responses, with many patients experiencing long-term remission. As a result, immune checkpoint inhibitors are increasingly being integrated into standard treatment regimens for HL, with ongoing research aimed at refining their use and minimizing side effects. CAR-T therapy has also emerged as a groundbreaking treatment, particularly in pediatric B-cell malignancies such as acute lymphoblastic leukemia and B-cell NHL. CAR-T therapy involves genetically modifying a patient’s T-cells to express a receptor that recognizes and targets cancer cells. The success of CAR-T therapy in treating relapsed or refractory pediatric lymphomas has been a major milestone. The therapy has led to high rates of complete remission in some children who would otherwise have had limited treatment options. However, the use of CAR-T therapy is not without risks, such as cytokine release syndrome and neurotoxicity, which require careful monitoring. The ongoing refinement of CAR-T therapies, including the development of next-generation CAR-T cells with enhanced targeting and reduced side effects, is likely to expand their utility in pediatric LPDs.^[39,40]^

### 13.3. Personalized medicine and targeted therapies

The shift toward personalized medicine is another emerging trend in the treatment of pediatric LPDs. With the increasing availability of genomic sequencing technologies, clinicians can now tailor treatment based on the individual genetic and molecular profile of a patient’s disease. This approach allows for more precise targeting of therapy, improving efficacy while reducing unnecessary toxicity. In addition to immune checkpoint inhibitors and CAR-T therapy, other forms of targeted therapy are under investigation for pediatric LPDs. For instance, small molecule inhibitors targeting the PI3K/AKT/mTOR pathway, which is frequently dysregulated in lymphoid malignancies, are being explored in clinical trials. Similarly, drugs that target the BCL-2 family of proteins, which regulate cell survival, are showing promise in the treatment of B-cell lymphomas. These therapies offer the potential to disrupt key survival mechanisms within tumor cells, leading to improved outcomes in patients with resistant disease. Furthermore, the identification of genetic mutations and chromosomal translocations in pediatric LPDs is informing the development of therapies that specifically target these abnormalities. For example, inhibitors of the JAK/STAT signaling pathway, which are being investigated in both HL and NHL, hold great promise for future use, particularly in cases that involve these molecular alterations.^[50,53–55]^

### 13.4. Role of the microenvironment in disease progression

Recent studies have increasingly highlighted the role of the tumor microenvironment (TME) in the pathogenesis of pediatric lymphomas. The TME consists of a complex network of immune cells, stromal cells, and extracellular matrix components that interact with tumor cells and play a critical role in disease progression and treatment resistance. In HL, the Reed–Sternberg cells secrete cytokines and chemokines that recruit immune cells into the tumor microenvironment, creating a niche that supports tumor growth while suppressing anti-tumor immune responses. Understanding the dynamics of the TME in pediatric LPDs is leading to the development of novel strategies that aim to modulate the immune landscape to favor anti-tumor immunity. For example, targeting the immune checkpoint molecules expressed within the TME, such as PD-L1, or reprogramming the TME to increase T-cell infiltration into the tumor, is being actively investigated as a potential therapeutic strategy.^[56–58]^

### 13.5. Minimizing long-term toxicity

As survival rates for pediatric LPDs continue to improve, there is an increasing focus on minimizing the long-term toxicity of treatments. Traditional chemotherapy and radiation therapies, while effective in treating LPDs, can result in significant late effects, including secondary cancers, endocrine disorders, and cardiovascular complications. As a result, there is a growing emphasis on developing less toxic treatment regimens that provide effective disease control while minimizing these risks. For example, in HL, reducing the dose and field of radiation therapy, particularly in young patients, has become a key strategy to lower the risk of secondary malignancies. Additionally, the use of less toxic chemotherapy regimens, such as those that incorporate lower doses of alkylating agents, is being explored. The integration of targeted therapies and immunotherapies into treatment protocols offers the potential to achieve effective disease control with fewer long-term side effects.^[59]^

### 13.6. The role of early detection and biomarkers

Early detection and the use of biomarkers are crucial in improving the prognosis for pediatric LPDs. Advances in molecular imaging techniques, such as PET scans, and the identification of ctDNA are allowing for more accurate staging and monitoring of disease. ctDNA, in particular, has the potential to serve as a noninvasive biomarker for minimal residual disease and relapse, enabling clinicians to detect relapses earlier and adjust treatment accordingly.^[60,61]^

## 14. Challenges and future directions

Despite significant advancements in the understanding and management of pediatric LPDs, several challenges persist. The complexity and heterogeneity of these diseases, coupled with the evolving nature of treatment approaches, require continuous refinement and adaptation of current strategies. This section outlines the major challenges in the management of pediatric LPDs and explores the future directions that hold promise for overcoming these obstacles and improving patient outcomes.

### 14.1. Diagnostic challenges and disease heterogeneity

One of the primary challenges in the management of pediatric LPDs is the difficulty in diagnosing these disorders early and accurately. LPDs in children can present with symptoms that overlap with other more common pediatric illnesses, leading to delays in diagnosis. The heterogeneity of LPDs, ranging from benign, self-limited reactive conditions to aggressive, life-threatening malignancies, further complicates the diagnostic process. Molecular profiling and genetic testing have revolutionized the diagnostic landscape; however, these technologies are not yet universally accessible, particularly in resource-limited settings. The reliance on advanced diagnostic techniques such as NGS and immunohistochemistry is still a challenge due to high costs and the need for specialized expertise. There is a need for the development of cost-effective, high-throughput diagnostic tools that can be implemented in various clinical settings to enhance early detection and improve prognostic stratification.^[30]^

### 14.2. Limited therapeutic options for rare subtypes

Pediatric LPDs encompass a wide range of subtypes, many of which are rare and lack established treatment protocols. For example, certain forms of B-cell and T-cell lymphomas in children, as well as EBV-associated LPDs, present unique challenges in terms of treatment due to the rarity of these conditions and the lack of comprehensive clinical trials. Although the advent of immunotherapies, such as immune checkpoint inhibitors and CAR-T cell therapies, has been transformative in treating some pediatric lymphomas, their application remains limited to specific subtypes and resistant cases. For rarer or more resistant forms of LPDs, effective treatment options are still lacking. Additionally, many of these therapies are still undergoing clinical trials, and their long-term safety and efficacy, particularly in children, remain uncertain. Expanding the use of targeted therapies and immunotherapies to address the full spectrum of pediatric LPD subtypes is an ongoing challenge that requires further research.^[62]^

### 14.3. Long-term toxicity and quality of life

As the survival rates for pediatric LPDs continue to improve, a new challenge has emerged: the management of long-term toxicity from current treatment regimens. Traditional therapies, such as chemotherapy and radiation, are highly effective but can cause significant long-term side effects, including secondary cancers, cognitive impairments, cardiovascular diseases, and endocrine disorders. The focus of treatment has gradually shifted from achieving survival to also ensuring that survivors can lead healthy, productive lives. The development of less toxic therapies, particularly those that minimize the use of chemotherapy and radiation, is a key priority. The use of targeted therapies and immunotherapies offers hope in this area, but their long-term side effects are still being studied. Therefore, the challenge lies in balancing the efficacy of aggressive treatments with their potential to cause lasting harm. The future of pediatric LPD management must focus on reducing treatment-related toxicities while maintaining effective disease control.^[58]^

### 14.4. Drug resistance and relapse

Although many children with LPDs achieve remission, relapse remains a significant challenge, particularly in cases where the disease is refractory to treatment. The mechanisms underlying drug resistance in pediatric LPDs are not yet fully understood, but they likely involve genetic mutations, alterations in the tumor microenvironment, and immune evasion strategies. For instance, in the case of NHL, resistance to chemotherapy agents such as cyclophosphamide and doxorubicin is a well-known challenge. Similarly, relapse in pediatric HL, even after successful chemotherapy and radiation, remains a serious concern. Identifying biomarkers of resistance and developing novel agents that target these mechanisms could significantly improve long-term outcomes for pediatric LPD patients.^[63]^

### 14.5. Access to care and global health disparities

Another critical challenge in the management of pediatric LPDs is the disparity in access to care across different regions of the world. In high-income countries, advances in molecular diagnostics, immunotherapy, and supportive care have improved survival rates, but in low- and middle-income countries (LMICs), access to these innovations is often limited. Resource constraints, including the lack of specialized healthcare infrastructure, trained healthcare personnel, and expensive medications, contribute to these disparities. In particular, the cost of advanced therapies like CAR-T cell therapy and immune checkpoint inhibitors remains prohibitively high. As a result, many children in resource-limited settings may not receive timely and appropriate treatment, leading to poorer outcomes. Addressing these global health disparities will require not only the development of affordable treatments but also the establishment of systems to support early diagnosis, equitable access to therapies, and long-term care for survivors.^[64]^

### 14.6. Personalizing treatment for diverse pediatric populations

The field of pediatric LPDs is moving towards more personalized treatment strategies based on the molecular characteristics of each patient’s disease. However, personalizing treatment in pediatric patients presents unique challenges due to the variability of responses to therapies and the developmental aspects of the pediatric immune system. Children are not simply “small adults”; their immune systems and disease biology differ significantly from those of adults, which affects how they respond to treatment. The challenge lies in identifying biomarkers that can predict treatment response, minimize toxicity, and guide therapeutic decisions. Furthermore, the fact that pediatric patients are still growing and developing means that therapies that are safe and effective in adults may not be appropriate for children. Future research must focus on developing treatment protocols that are specifically tailored to pediatric patients, accounting for their unique physiological and immunological characteristics.^[65]^

#### 14.6.1. Future directions

Despite the challenges, several promising avenues are emerging that may address these issues and improve outcomes for pediatric LPDs.

1. Expanding the role of immunotherapies: the continued development of immune checkpoint inhibitors, CAR-T cell therapies, and bispecific T-cell engagers holds great promise in treating pediatric LPDs, particularly those that are resistant to traditional therapies. Ongoing clinical trials aimed at expanding the indications for these therapies and improving their safety profiles are critical to the future of pediatric lymphoma treatment.^[40]^
2. Biomarkers and personalized medicine: the development of better biomarkers for early detection, risk stratification, and monitoring of minimal residual disease will be crucial. Additionally, personalized treatment strategies based on the genetic and molecular profiles of individual patients will allow for more precise targeting of therapies, minimizing side effects while improving efficacy.^[66]^
3. Improving access to care: addressing the disparities in access to care across different regions of the world is critical. Efforts to reduce the cost of advanced treatments, improve diagnostic capabilities, and enhance training for healthcare providers in resource-limited settings will be essential for improving global outcomes in pediatric LPDs.^[65]^
4. Reducing long-term toxicities: ongoing research into less toxic therapies, including targeted therapies and immunotherapies, is needed to reduce the long-term sequelae of treatment. Additionally, developing better strategies for managing survivors, including regular monitoring for late effects and providing supportive care, will be key to improving the quality of life for pediatric LPD patients.^[58]^

## 15. Recommendations in pediatric LPDs management

The management of pediatric LPDs is complex, requiring a multi-faceted approach to improve diagnostic accuracy, treatment efficacy, and patient outcomes. Based on the challenges identified in the management of these conditions, several key recommendations can be made to address current gaps in care and pave the way for future advancements in the field.

### 15.1. Enhancing early diagnosis and accurate classification

One of the most critical steps in improving outcomes for pediatric LPD patients is enhancing the early diagnosis and accurate classification of these disorders. Given the heterogeneity of LPDs, it is essential to integrate advanced molecular diagnostic techniques into routine clinical practice. Genomic profiling, including NGS, should be more widely adopted to identify genetic mutations and other biomarkers associated with these disorders. Additionally, standardizing the use of immunohistochemistry and flow cytometry for subtype classification would enable more accurate diagnosis and risk stratification. Moreover, raising awareness and educating healthcare providers, particularly in resource-limited settings, on the subtle clinical presentations of pediatric LPDs could aid in earlier identification. This could be achieved through continuous professional development programs, diagnostic workshops, and international collaborations to share knowledge and best practices.

### 15.2. Expanding access to advanced therapies

While recent advancements in immunotherapy, including CAR-T cell therapy and immune checkpoint inhibitors, have shown great promise in treating pediatric lymphomas, these treatments remain inaccessible to many due to high costs and logistical challenges. One of the most pressing recommendations is to work toward making these therapies more affordable and accessible to a broader patient population. This can be achieved by advocating for policy changes, promoting public-private partnerships, and encouraging pharmaceutical companies to engage in cost-reduction strategies or offer treatments through subsidized programs. Furthermore, there is a need for the development of lower-cost alternatives, such as biosimilars and generics, to expand access to these therapies. Strengthening the healthcare infrastructure in LMICs to support the delivery of advanced therapies will be essential in addressing global health disparities.

### 15.3. Fostering personalized medicine approaches

The future of pediatric LPD management lies in personalized medicine, where treatment is tailored to the specific molecular and genetic characteristics of each patient’s disease. It is essential to integrate precision medicine into clinical protocols, ensuring that patients receive therapies best suited to their individual profiles. This requires further investment in molecular research to identify novel biomarkers for disease progression, therapeutic response, and relapse risk. Additionally, expanding clinical trials to include diverse pediatric populations, including those from LMICs, will ensure that personalized approaches are developed with a global perspective. Collaboration across research institutions, academia, and pharmaceutical companies is key to advancing the field of personalized treatment and identifying novel therapeutic targets.

### 15.4. Addressing long-term toxicity and survivorship care

As survival rates for pediatric LPDs improve, the focus must shift toward managing long-term toxicities associated with current treatment regimens, such as chemotherapy and radiation. Research should focus on identifying and developing therapies that minimize the long-term side effects of chemotherapy and radiation. Targeted therapies, immunotherapies, and less aggressive chemotherapy regimens offer potential for reducing toxicities. Establishing comprehensive survivorship programs that monitor for late effects of treatment is crucial. These programs should include regular screenings for secondary cancers, cardiovascular diseases, endocrine dysfunctions, and cognitive impairments. Collaborating with multidisciplinary teams, including cardiologists, endocrinologists, and neurologists, will help in providing holistic care for survivors. Psychological and emotional support for survivors and their families should be integrated into survivorship programs to address the emotional burden of living with the long-term effects of cancer treatment.

### 15.5. Increasing collaborative global research

Given the global disparities in pediatric LPD outcomes, fostering collaborative international research is essential. Increased investment in multi-center clinical trials that include diverse patient populations is needed to better understand the epidemiology, biology, and outcomes of pediatric LPDs in different regions of the world. Moreover, partnerships between high-income countries and LMICs should be strengthened to share resources, knowledge, and clinical expertise. Building research capacity in LMICs, through initiatives such as training programs and collaborative research networks, will help bridge the gap in understanding and managing pediatric LPDs globally.

### 15.6. Raising awareness and educating communities

A critical aspect of improving outcomes for pediatric LPDs involves raising awareness among both the general public and healthcare providers. Educational campaigns about the signs and symptoms of pediatric LPDs can facilitate early detection, while training healthcare providers, especially in under-resourced regions, will ensure timely diagnosis and appropriate treatment. Local healthcare teams should be supported with ongoing education, so they are equipped with the latest knowledge and skills to manage pediatric LPDs. Collaboration between international health organizations, local governments, and non-governmental organizations could play a significant role in these efforts.

### 15.7. Strengthening pediatric LPD networks and support systems

It is essential to strengthen networks for pediatric LPD patients and their families. These support systems, which can be facilitated through patient advocacy groups, can provide invaluable emotional and practical support for families navigating the challenges of diagnosis and treatment. In addition, these networks can promote the sharing of experiences and information, helping families find resources and support, as well as increasing awareness of clinical trials and emerging therapies.

## 16. Conclusion

Pediatric LPDs present significant challenges in both diagnosis and treatment due to their diverse nature and the complexity of the pediatric immune system. While advancements in molecular diagnostics and immunotherapies have improved outcomes, gaps remain in the effective management of rare and refractory forms of LPDs, as well as in reducing long-term toxicities. The limited availability of personalized treatment options and the disparities in access to advanced care, particularly in low-resource settings, further complicate the landscape of pediatric LPDs.

## Author contributions

**Conceptualization:** Emmanuel Ifeanyi Obeagu.

**Methodology:** Emmanuel Ifeanyi Obeagu.

**Resources:** Emmanuel Ifeanyi Obeagu.

**Supervision:** Emmanuel Ifeanyi Obeagu.

**Validation:** Emmanuel Ifeanyi Obeagu.

**Visualization:** Emmanuel Ifeanyi Obeagu.

**Writing – original draft:** Emmanuel Ifeanyi Obeagu.

**Writing – review & editing:** Emmanuel Ifeanyi Obeagu.
